# Diabetes, hyperglycemia, and brain MRI biomarkers: results from SOL-INCA MRI study

**DOI:** 10.1038/s41387-026-00415-z

**Published:** 2026-04-07

**Authors:** Kevin A. González, Wassim Tarraf, Sarah J. Banks, Katherine J. Bangen, Judy Pa, Linda C. Gallo, Ariana M. Stickel, Paola Filigrana, Carmen R. Isasi, Martha Daviglus, Fernando D. Testai, Melissa Lamar, Charles DeCarli, Hector M. González

**Affiliations:** 1 Department of Neurosciences, University of California, San Diego School of Medicine, San Diego, CA USA; 2 Department of Healthcare Sciences and Institute of Gerontology, Wayne State University, Detroit, MI USA; 3 Department of Psychiatry, University of California, San Diego School of Medicine, San Diego, CA USA; 4 VA San Diego Healthcare System, San Diego, CA USA; 5 Department of Psychology and South Bay Latino Research Center, San Diego State University, San Diego, CA USA; 6 Department of Epidemiology and Population Health, Albert Einstein College of Medicine, Bronx, NY USA; 7 Institute for Minority Health Research, University of Illinois Chicago, College of Medicine, Chicago, IL USA; 8 Department of Neurology and Rehabilitation, University of Illinois Chicago, Chicago, IL USA; 9 Rush Alzheimer’s Disease Research Center, Rush University Medical Center, Chicago, IL USA; 10 Department of Neurology, University of California, Davis, Sacramento, CA USA

**Keywords:** Risk factors, Epidemiology

## Abstract

**Objective:**

Hispanic/Latino individuals have higher rates of type 2 diabetes and Alzheimer’s Disease and Related Dementia (ADRD) burden compared to non-Hispanic whites. Diabetes is a risk factor for ADRD, but the extent of its associations with brain markers in community-dwelling Hispanic/Latino individuals is unknown. We examined how glycemic dysregulation and diabetes associate with small vessel disease damage and neurodegeneration in Hispanic/Latino adults from a large and community-representative cohort study.

**Research design and methods:**

We used data from 2627 individuals, aged 35–85 years, from the Hispanic Community Health Study/Study of Latinos (HCHS/SOL; 2008–2011) who underwent brain imaging through the SOL/Investigations of Neurocognitive Aging MRI (SOL-INCA MRI; 2018–2022) study. Exposures included diabetes status and HbA1c (%) levels. Outcomes included white matter hyperintensities, free water, fractional anisotropy, and volumetric regions including hippocampus, lateral ventricles, total brain, and cortical gray matter.

**Results:**

Diabetes status, compared to no diabetes, was associated with larger white matter hyperintensity volume, lower fractional anisotropy, and higher free water. Diabetes status was also associated with larger lateral ventricles as well as smaller total brain, frontal gray matter, and occipital gray matter volumes. The association between diabetes and brain MRI outcomes was stronger in middle-aged and older individuals (50 years and older) compared to younger individuals (35–49 years).

**Conclusion:**

Diabetes was associated with markers of small vessel disease (white matter micro and macrostructural damage) and neurodegeneration (smaller brain volumes). White matter hyperintensities have been associated with increased risk of stroke and cognitive decline. Other work has found that free water and fractional anisotropy may predict worse cognitive performance, even in normal-appearing white matter. Smaller brain volumes have also been associated with cognitive deficits. These findings highlight the additional ADRD burden this population faces due to their higher diabetes prevalence.

## Introduction

Diabetes disproportionally affects Hispanic/Latino communities compared to non-Hispanic whites, with around 6.4 million Hispanic/Latino individuals (15% of the population) in the US having diabetes [1]. Nearly 2 in 5 Hispanic/Latino individuals with diabetes may be undiagnosed, likely due to low levels of health insurance and healthcare use [2]. The etiology of diabetes in Hispanic/Latinos is multifaceted and is impacted by sociocultural, economic, lifestyle, and genetic factors [3]. Diabetes is also a major risk factor for Alzheimer’s Disease and Related Dementias (ADRD) [4, 5]. Despite higher prevalence of diabetes and higher exposure to its brain sequalae as a result of underdiagnosis and undertreatment, Hispanic/Latino communities remain understudied in ADRD research. Examining how diabetes affects brain outcomes is critical for reducing health disparities and ADRD burden in this high-risk population.

Diabetes affects the microvasculature [6] and increases ADRD burden through vascular processes (e.g., small vessel disease) [7]. Hyperglycemia and hyperinsulinemia could damage endothelial cells and pericytes, both critical components of the neurovascular system. These vascular lesions may lead to white matter damage [6, 8]. White matter hyperintensities (WMHs), fractional anisotropy (FA), and free water (FW) are common markers of white matter damage and have been associated with small vessel disease (SVD) and ischemic stroke [8, 9]. Diabetes has been associated with increased WMHs and lower FA [10]; however, less work has examined associations of diabetes and FW, particularly less so in Hispanic/Latino populations. Some studies, like the HABLE study, found that elevated hemoglobin A1c (HbA1c) was associated with more WMH volume in Mexican individuals but not in white participants [11]. Notably, we have found that volumetric MRI measures differ within Hispanic/Latino individuals, which suggests these associations could vary depending on background/heritage [12]. These results highlight the need of further work in diverse Hispanic/Latino populations, especially those that include measures such as FW and FA to get a more comprehensive understanding of white matter micro and macrostructure.

Diabetes might also accelerate neurodegeneration and brain atrophy [13]; diabetes has been associated with larger lateral ventricles, smaller gray matter volumes, and smaller hippocampal volumes [14, 15]. Although SVD is a risk factor for ADRD [16], some evidence suggests that diabetes may lead to neurodegeneration through non-vascular mechanisms [15, 17]. De la Monte et al. (2015), for example, proposed that brain insulin resistance may lead to neurodegeneration through molecular processes that promote oxidative stress, apoptosis, and increased production of hyperphosphorylated tau and amyloid-β [18]. Hyperglycemia could also promote neural loss and axonal damage through oxidative pathways that are upregulated by increased glucose concentration [19]. Thus, it is critical to examine both vascular (e.g., SVD) and non-vascular (e.g., neurodegeneration) brain outcomes in the context of diabetes, as they may underlie distinct processes.

In this study, we aim to examine the associations between diabetes and MRI biomarkers of SVD and neurodegeneration. We hypothesize that diabetes status and elevated HbA1c % levels will be associated with (1) SVD biomarkers and (2) neurodegeneration markers. We also hypothesize that the association between diabetes and magnetic resonance imaging (MRI) outcomes will be stronger in older individuals (ages 50+) compared to younger individuals (ages 35–49). We believe that is the case because older individuals should, on average, have been exposed to diabetes longer than younger individuals.

## Methods

### Dataset

We used data from the Hispanic Community Health Study/Study of Latinos (HCHS/SOL, 2008–2011), a prospective study of diverse Hispanic/Latino communities (Central American, Cuban, Dominican, Mexican, Puerto Rican, South American). HCHS/SOL recruited *n* = 16,415 individuals ages 18–74 across four testing centers: Bronx, NY; Chicago, IL; Miami, FL; San Diego, CA. Blood samples were assayed for vascular factors (e.g., HbA1c). More information about HCHS/SOL study methods and design can be found at the HCHS/SOL website: https://sites.cscc.unc.edu/hchs and was published elsewhere [20]. All four testing centers received institutional review board approvals. All individuals who participated in the study provided informed written consent in their preferred language.

The Study of Latinos-Investigations of Neurocognitive Aging (SOL-INCA, 2016–2018) is an ancillary study of HCHS/SOL. During the second HCHS/SOL visit (years 2015–2018), the SOL-INCA study recruited *n* = 6377 from the parent HCHS/SOL study. The goal of SOL-INCA is to study neurocognitive aging in diverse Hispanic/Latino populations [21]. A subsample of SOL-INCA (ages 50+, *n* = 2391) HCHS/SOL (ages 35–49, *n* = 276) individuals underwent brain imaging through the SOL-Investigation of Neurocognitive Aging MRI ancillary study (SOL-INCA MRI), on average 10 years later (range: 7–14 years). The SOL-INCA MRI study included individuals ages 18–49 to obtain a more comprehensive adult lifespan perspective of neurocognitive health and potentially detect earlier markers of neurostructural damage. A full derivation flowchart is available in Supplementary Fig. S1. The goal of the SOL-INCA MRI study is to examine brain health and neurobiological outcomes in middle-aged and older Hispanic/Latinos (50+ years at time of MRI). SOL-INCA MRI combines both volumetric and microstructural measures to characterize both non-vascular and vascular brain pathology to further understand Alzheimer’s Disease risk. SOL-INCA MRI was weighted to allow for generalizable findings relative to the HCHS/SOL target population in the four geographic areas of focus. The final sample of individuals (including those ages 50+ and 35–50) who underwent imaging and had processed MRI data includes *n* = 2673 (weighted: 56% women, 43% men).

### Analytic sample

A total unweighted *n* = 2673 individuals had available MRI data at the time of this study, but *n* = 46 individuals were excluded based on missingness in our covariates for a final unweighted sample of *n* = 2627. In stratified analysis, we examined two subgroups, including middle-aged and older individuals (ages 50+; *n* = 2372) and younger individuals (ages 35–50; *n* = 255).

*MR imaging* was performed using 3 T MRI scanners: GE 3T 750 (three sites) and Philips 3T Achieve TX (one site). The sequences used in this analysis include: (1) T1 volume (1 mm^3^ resolution), (2) 3DFLAIR, (3) T2*GRE (gradient echo; 2D, long T2*GRE), and (4) Diffusion Tensor Imaging (DTI). The images were processed using protocols from the Imaging of Dementia and Aging (IDeA) laboratory at UC Davis. All images were visually checked at every segmentation step to ensure quality and to fix small errors (e.g., in skull removal or WMHs segmentation).

### MR processing

Detailed methodology on MR processing can be found in supplementary Methods S1. All MR processing was performed by the IDeA laboratory (UC Davis). At each step of the pipeline, all images are visually checked for minor errors (e.g., skull removal or WMH segmentation), and original images are available for reporting purposes. First, we removed non-brain sections in images using a skull stripping pipeline developed by Fletcher [22]. Next, we performed image intensity corrections to account for differences in image intensities across individuals [23]. An Expectation-Maximization (EM) approach was used to estimate gray matter volumes [24]. We estimated hippocampal volume using a mask developed by AEDC-ADNI group [25]. WMHs volume was calculated using a probabilistic model on FLAIR and 3D T1 images [26]. Lastly, DTI (FA and FW) measures were estimated using the FMRIB Software Library Toolkit [27].

*Outcomes* included both volumetric and microstructural metrics. Structural brain volumes were measured in cm^3^ and included total brain, combined gray matter (sum of 4 gray matter regions), frontal gray matter, occipital gray matter, temporal gray matter, parietal gray matter, lateral ventricles, WMHs, and hippocampus. Diffusion metrics included FA and FW and are represented as a scalar fraction (0–1). All measures, except for FA and FW, were residualized for total cranial volume and standardized (Z-scored). Lateral ventricles and WMHs volumes were log-transformed before residualization to account for non-normal distributions. The distributions of both measures were log-normal after transformation (Supplementary Fig. 3).

### Exposures

The main exposures of interest were diabetes status (no diabetes, prediabetes, diabetes) and continuous HbA1c levels measured at visit 1. Diabetes status was assessed using American Diabetes Association criteria [28], which accounts for glucose levels, diabetes medication use, and HbA1c % levels. Individuals were classified as having diabetes if they met any of the following criteria: (1) fasting glucose ≥126 mg/dL and fasting time > 8 h, (2) fasting glucose ≥200 mg/dL and fasting time < 8 h, (3) post-OGTT glucose ≥200 mg/dL, (4) HbA1c ≥ 6.5%, or (5) diabetes medication use. Prediabetes was classified as follows: (1) fasting glucose between 100 and 125 mg/dL and fasting time >8 h, (2) post-OGTT glucose between 140–199 mg/dL, or (3) HbA1c between 5.7 and 6.5%. We also used HbA1c % as a continuous exposure. HbA1c is a marker for glucose levels and insulin resistance and is an average of glucose levels across several weeks. *Covariates* included continuous age at MRI, sex (male, female), Hispanic/Latino background (Dominican, Puerto Rican, South American, Mexican, Central American, Cuban), education (less than high school, high school, more than high school), continuous body mass index (BMI), testing center (San Diego, Bronx, Chicago, Miami) and physical activity intensity (inactive, low activity, medium activity, high activity, based on the global physical activity questionnaire [29]). Except for age, all covariates were assessed at visit 1. Additionally, we adjusted for time between HCHS/SOL and SOL-INCA MRI visit (in years) in models using HbA1c % to account for the time lag between baseline laboratory testing (HbA1c) and MRI acquisition (on average, 10 years later).

**Fig. 1:**
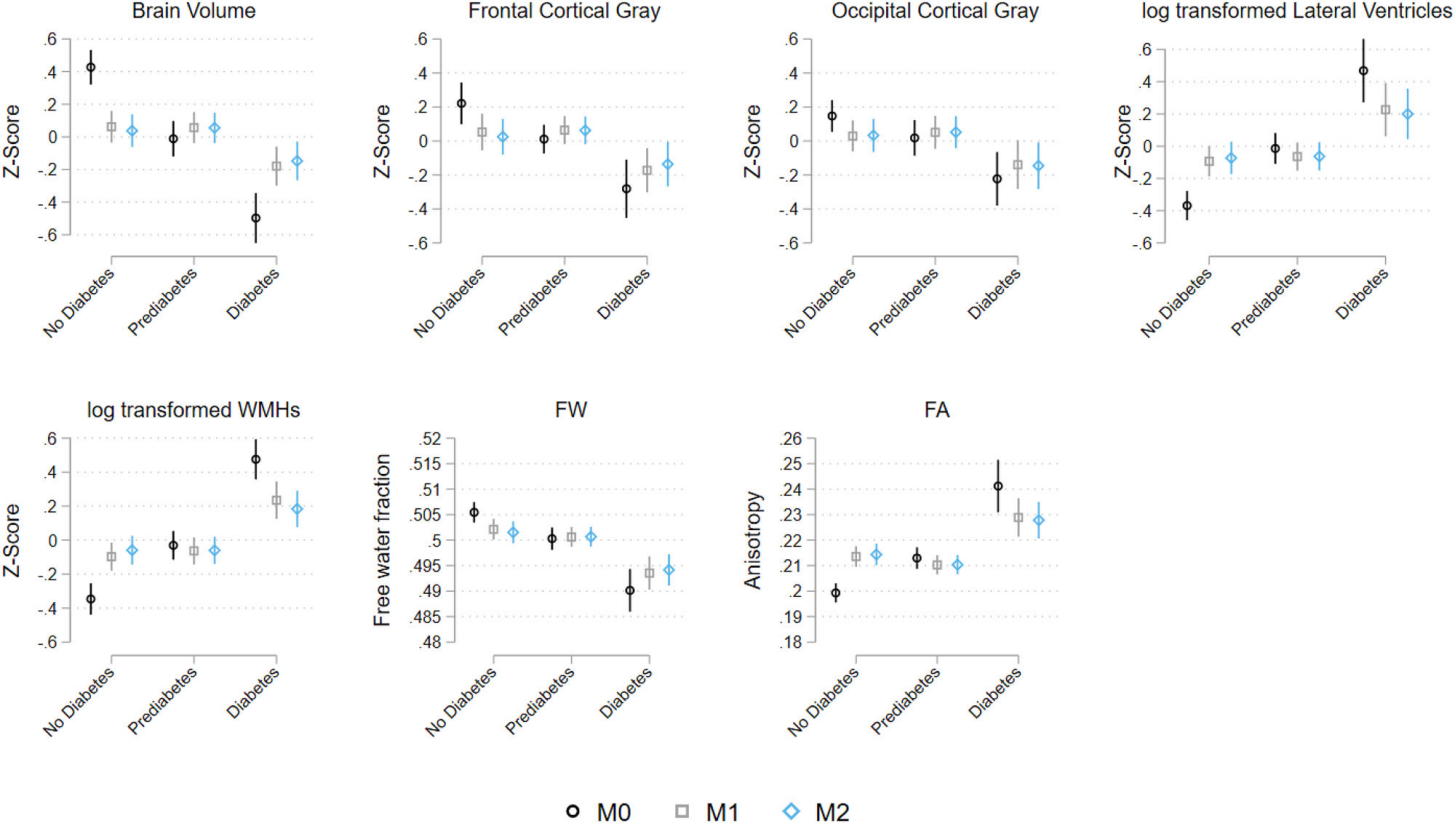
Associations between diabetes status and brain outcomes (marginal estimates and 95% confidence intervals, *N* = 2627). WMH white matter hyperintensities, FA fractional anisotropy, FW free water; all measures, except for FA and FW, are standardized and residualized for cranial volume. Lateral ventricles and WMHs were additionally log-transformed before residualization. *M0* model adjusts for time between visits. *M1* model includes continuous age, sex (Male, Female), Hispanic/Latino background (South American, Central American, Mexican, Cuban, Puerto Rican, and Dominican), and a trichotomous indicator for education (less than high school, high school or equivalent, more than high school). *M2* additionally includes a categorical measure for testing center (San Diego, Miami, Chicago, Bronx), continuous body mass index, and a four-category indicator for physical activity (Inactive, low activity, medium activity, high activity).

*Analysis* was generated using Stata 18 software. The analysis accounted for SOL-INCA MRI complex survey design and includes survey weights, which allow us to generalize findings to the target population (four major metropolitan cities). First, we generated descriptive statistics of our target population by age at MRI (Table 1). We tested differences across groups using analysis of variance (ANOVA) and chi-squared tests for continuous and categorical measures, respectively. To test the association between diabetes status or HbA1c % and brain outcomes, we conducted multivariable survey weighted linear models with (M0) adjustment for time between visits, (M1) age, sex, background, education, and testing center adjustments, and (M2) full covariate adjustments. We reported beta estimates and 95% CI intervals as measures of association for both diabetes status and continuous HbA1c % (Tables 2 and 3). Beta estimates between age and brain outcomes were also extracted and compared against beta estimates from exposure to provide a comparison with age-related equivalent change. Estimates of age (at MRI) equivalence were derived by dividing the beta estimate for diabetes exposure with the outcome by the beta estimate of age and the outcome. Lastly, we plotted estimates from models of the association between diabetes status and brain markers to improve readability of findings (Fig. 1). Plotted estimates for continuous HbA1c % can be found in Supplementary Fig. S2. We reported unweighted median and interquartile range of both BMI and HbA1c % in Supplementary Table S1.

**Table 1:** Descriptive statistics by age.

	<50 years	50+ years	Total	*P* value
**Unweighted** ***N***	255	2372	2627	
%	13.9	86.1	100	
**BMI mean (SD)**	30.12 [28.97; 31.28]	29.75 [29.44; 30.06]	29.80 [29.49; 30.11]	*p* = 0.667
**HbA1c %**	5.51 [5.36; 5.67]	6.08 [5.99; 6.17]	6.00 [5.92; 6.08]	*p* < 0.001
**Women % (SE)**	41.84 [35.28; 48.40]	58.14 [54.38; 61.90]	55.88 [52.59; 59.16]	*p* < 0.001
**Center**				
Bronx	34.56 [26.34; 42.78]	25.87 [22.25; 29.50]	27.08 [23.57; 30.59]	*p* = 0.008
Chicago	16.48 [11.61; 21.36]	11.99 [9.90; 14.08]	12.61 [10.53; 14.70]	
Miami	24.58 [17.40; 31.75]	37.90 [32.25; 43.56]	36.06 [30.77; 41.34]	
San Diego	24.38 [16.66; 32.10]	24.23 [20.03; 28.43]	24.25 [20.40; 28.11]	
**Education**				
Less than High School	29.80 [22.62; 36.99]	35.99 [32.46; 39.51]	35.13 [31.92; 38.33]	*p* = 0.007
High School or equivalent	29.81 [22.14; 37.48]	18.95 [16.63; 21.28]	20.46 [18.15; 22.77]	
Greater than High School	40.39 [32.40; 48.37]	45.06 [41.49; 48.62]	44.41 [41.11; 47.72]	
**Physical activity**				
Inactive	19.71 [13.83; 25.58]	26.76 [23.28; 30.24]	25.78 [22.56; 29.01]	*p* = 0.149
Low activity	13.89 [8.71; 19.06]	14.23 [11.78; 16.68]	14.18 [11.99; 16.38]	
Medium activity	10.85 [6.07; 15.63]	12.36 [10.17; 14.54]	12.15 [10.20; 14.09]	
High activity	55.55 [48.34; 62.76]	46.65 [43.10; 50.21]	47.89 [44.56; 51.21]	
**Hispanic/Latino background**				
Dominican	11.22 [5.37; 17.06]	8.17 [6.47; 9.87]	8.59 [6.73; 10.45]	*p* = 0.010
Central American	5.59 [2.76; 8.42]	7.83 [6.10; 9.56]	7.52 [5.89; 9.15]	
Cuban	17.27 [11.02; 23.51]	27.16 [22.18; 32.14]	25.79 [21.23; 30.35]	
Mexican	43.46 [34.95; 51.97]	30.81 [26.76; 34.87]	32.57 [28.69; 36.45]	
Puerto Rican	14.04 [8.69; 19.39]	16.15 [13.61; 18.68]	15.86 [13.47; 18.24]	
South American	2.71 [1.11; 4.31]	6.37 [4.78; 7.96]	5.86 [4.48; 7.24]	
More than one	5.71 [0.26; 11.17]	3.51 [2.18; 4.84]	3.82 [2.44; 5.19]	
**Diabetes status**				
No diabetes	62.02 [54.32; 69.72]	25.21 [22.80; 27.61]	30.31 [27.82; 32.81]	*p* < 0.001
Prediabetes	29.75 [22.62; 36.88]	46.72 [43.15; 50.29]	44.37 [41.19; 47.54]	
Diabetes status	8.23 [2.68; 13.79]	28.07 [24.25; 31.89]	25.32 [21.87; 28.78]	

Diabetes status was ascertained using ADA guidelines.

Mean and standard deviation (in parentheses) were presented for continuous measures.

Prevalence and standard error (in parentheses) were presented for categorical measures.

*HbA1c* Hemoglobin A1C, *BMI* body mass index, *SE* standard error, *SD* standard deviation.

**Table 2:** Associations between diabetes status and brain outcomes (*N* = 2627).

	Total brain		Combined gray		Frontal gray	
	b [95% CI]	b [95% CI]	b [95% CI]	b [95% CI]	b [95% CI]	b [95% CI]
	M1	M2	M1	M2	M1	M2
**No diabetes**	ref	ref	ref	ref	ref	ref
**Prediabetes**	−0.007 [−0.100; 0.086]	0.006 [−0.085; 0.096]	0.042 [−0.061; 0.145]	0.054 [−0.045; 0.153]	0.019 [−0.088; 0.126]	0.032 [−0.072; 0.136]
**Diabetes**	−0.248*** [−0.360; −0.136]	−0.218*** [−0.330; −0.107]	−0.161* [−0.300; −0.021]	−0.130 [−0.266; 0.006]	−0.212** [−0.346; −0.079]	−0.179** [−0.311; −0.047]
**Age decline**^a^	4.2 years	3.7 years	3.9 years	3.2 years	7.5 years	6.3 years
	**Occipital gray**		**Temporal gray**		**Parietal gray**	
	**b [95% CI]**	**b [95% CI]**	**b [95% CI]**	**b [95% CI]**	**b [95% CI]**	**b [95% CI]**
	**M1**	**M2**	**M1**	**M2**	**M1**	**M2**
**No diabetes**	ref	ref	ref	ref	ref	ref
**Prediabetes**	0.027 [−0.098; 0.152]	0.025 [−0.104; 0.154]	−0.013 [−0.117; 0.091]	−0.011 [−0.112; 0.089]	0.101 [−0.016; 0.218]	0.121* [0.007; 0.235]
**Diabetes**	−0.160* [−0.319; −0.002]	−0.163* [−0.325; −0.001]	0.061 [−0.068; 0.189]	0.066 [−0.065; 0.196]	−0.105 [−0.271; 0.062]	−0.060 [−0.221; 0.101]
**Age decline**^a^	7.3 years	7.5 years	−1.4 years	−1.6 years	4.0 years	2.3 years
	**Lateral ventricles**		**Hippocampus**		**WMHs**	
	**b [95% CI]**	**b [95% CI]**	**b [95% CI]**	**b [95% CI]**	**b [95% CI]**	**b [95% CI]**
	**M1**	**M2**	**M1**	**M2**	**M1**	**M2**
**No diabetes**	ref	Ref	ref	ref	ref	ref
**Prediabetes**	0.026 [−0.085; 0.137]	0.009 [−0.102; 0.121]	0.104 [−0.017; 0.226]	0.085 [−0.032; 0.202]	0.026 [−0.068; 0.120]	0.012 [−0.083; 0.106]
**Diabetes**	0.318*** [0.143; 0.493]	0.288*** [0.119; 0.457]	0.156 [−0.022; 0.335]	0.113 [−0.065; 0.291]	0.318*** [0.191; 0.445]	0.286*** [0.158; 0.413]
**Age decline**^a^	6.9 years	6.2 years	−6.3 years	−4.7 years	7.0 years	6.2 years
	**FA**		**FW**			
	**b [95% CI]**	**b [95% CI]**	**b [95% CI]**	**b [95% CI]**		
	**M1**	**M2**	**M1**	**M2**		
**No diabetes**	ref	ref	ref	ref		
**Prediabetes**	−0.002 [−0.004; 0.001]	−0.001 [−0.003; 0.001]	−0.003 [−0.007; 0.001]	−0.003 [−0.007; 0.001]		
**Diabetes**	−0.009*** [−0.012; −0.006]	−0.008*** [−0.011; −0.005]	0.016*** [0.009; 0.022]	0.016*** [0.010; 0.022]		
**Age decline**^a^	15.8 years	14.2 years	7.0 years	7.2 years		

*M1* model includes time between visits, continuous age, sex (Male, Female), Hispanic/Latino background (South American, Central American, Mexican, Cuban, Puerto Rican, and Dominican), a trichotomous indicator for education (less than high school, high school or equivalent, more than high school), and a categorical measure for testing center (San Diego, Miami, Chicago, Bronx).

*M2* additionally includes continuous body mass index, and a four-category indicator for physical activity (Inactive, low activity, medium activity, high activity).

*WMH* white matter hyperintensities, *FA* fractional anisotropy, *FW* free water.

*p < 0.05; **p < 0.01; ***p < 0.001. Differences relative to reference group (no diabetes).

^a^Age decline is calculated by dividing beta estimate from the diabetes group by the beta estimate for age from the same model. This provides us with an approximate measure of age equivalent change for diabetes exposure (e.g., for M2 total brain model, 1 year of age was associated with −0.059 standard deviation change in total brain volume. Diabetes was associated with −0.218 change: −0.218/−0.059 ≈ 3.7 year equivalent change).

In stratified models, we investigated differential outcomes based on individual age. We ran stratified linear models using a middle-aged and older cohort (ages 50+; Supplementary Table S2) younger individuals (ages 35–49; Supplementary Table S3). To account for possible confounders, we fit two extra models: one additionally adjusted for hypertension and smoking, the other adjusted for scanner type (Supplementary Table S4). We re-estimated the models using a four-category indicator for HbA1c % (<5.5%, 5.5–6.5%, 6.5–7%, >7%). These results are presented in Supplementary Table S5. Lastly, we plotted histograms of all the outcomes and plotted them against normal distributions (Supplementary Fig. S3).

## Results

*Descriptive* statistics by age (at MRI) can be found in Table 1. A total of *n* = 2627 individuals were included. The mean age at MRI was 64 years, and 86% of participants were 50 years or older. We found that individuals 50 and older were more likely to be women (58% women) compared to those 34–49 (42% women). One in four individuals met criteria for diabetes status (25%), and nearly half of all individuals (45%) met criteria for prediabetes. Median and interquartile range for continuous covariates can be found in Supplementary Table S1.

### Linear regression

Linear regression models can be found in Table 2 and Fig. 1. Compared to the no diabetes group, diabetes status was associated with smaller total brain (b = −0.218, [−0.330; −0.107], *p* < 0.001), frontal gray matter (b = −0.179, [−0.311; −0.047], *p* < 0.01), occipital gray matter (b = −0.163, [−0.325; −0.001], *p* < 0.05), larger lateral ventricles (b = 0.288, [0.119; 0.457], *p* < 0.001), increased WMHs volume (b = 0.286, [0.158; 0.413], *p* < 0.001), lower FA fraction (b = −0.009, [−0.012; −0.006], *p* < 0.001), and increased FW fraction (b = 0.016, [0.010; 0.022], *p* < 0.001). Prediabetes, compared to no diabetes, was associated with larger parietal cortical volume (b = 0.121, [0.007; 0.235], *p* < 0.05). These findings were consistent for HbA1c, with the additional association with larger hippocampal volume (Table 3 and Supplementary Fig. S2).

**Table 3:** Associations between continuous A1c and brain outcomes (*N* = 2627).

	Total brain		Combined gray	
	b [95% CI]	b [95% CI]	b [95% CI]	b [95% CI]
	M1	M2	M1	M2
HbA1c %	−0.070*** [−0.106; −0.034]	−0.063*** [−0.098; −0.027]	−0.047* [−0.083; −0.010]	−0.039* [−0.076; −0.003]
Age decline^a^	1.2 years	1.1 years	1.1 years	1.0 years
	**Frontal gray**		**Occipital gray**	
	**b [95% CI]**	**b [95% CI]**	**b [95% CI]**	**b [95% CI]**
	**M1**	**M2**	**M1**	**M2**
HbA1c %	−0.047** [−0.083; −0.012]	−0.039* [−0.075; −0.002]	−0.056** [−0.097; −0.015]	−0.056** [−0.097; −0.015]
Age decline^a^	1.6 years	1.3 years	2.5 years	2.5 years
	**Temporal gray**		**Parietal gray**	
		**b [95% CI]**	**b [95% CI]**	**b [95% CI]**
	**M1**	**M2**	**M1**	**M2**
HbA1c %	0.028 [−0.010; 0.067]	0.030 [−0.008; 0.068]	−0.058* [−0.104; −0.013]	−0.050* [−0.094; −0.006]
Age decline^a^	−0.7 years	−0.7 years	2.3 years	2.0 years
	**Lateral ventricles**		**Hippocampus**	
	**b [95% CI]**	**b [95% CI]**	**b [95% CI]**	**b [95% CI]**
	**M1**	**M2**	**M1**	**M2**
HbA1c %	0.097*** [0.051; 0.143]	0.089*** [0.046; 0.133]	0.058** [0.015; 0.101]	0.049* [0.007; 0.091]
Age decline^a^	2.1 years	1.9 years	−2.4 years	−2.0 years
	**WMH**		**FA**	
	**b [95% CI]**	**b [95% CI]**	**b [95% CI]**	**b [95% CI]**
	**M1**	**M2**	**M1**	**M2**
HbA1c %	0.089*** [0.049; 0.129]	0.080*** [0.041; 0.120]	−0.003*** [−0.004; −0.002]	−0.002*** [−0.004; −0.001]
Age decline^a^	1.9 years	1.7 years	4.4 years	4.0 years
	**FW**			
	**b [95% CI]**	**b [95% CI]**		
	**M1**	**M2**		
HbA1c %	0.005*** [0.002; 0.007]	0.005*** [0.002; 0.007]		
Age decline^a^	2.0 years	2.0 years		

*M1* model includes time between visits, continuous age, sex (Male, Female), Hispanic/Latino background (South American, Central American, Mexican, Cuban, Puerto Rican, and Dominican), a trichotomous indicator for education (less than high school, high school or equivalent, more than high school), and a categorical measure for testing center (San Diego, Miami, Chicago, Bronx).

*M2* additionally includes continuous body mass index, and a four-category indicator for physical activity (Inactive, low activity, medium activity, high activity).

*HbA1c* Hemoglobin A1c, *WMH* white matter hyperintensities, *FA* fractional anisotropy, *FW* free water.

*p < 0.05; **p < 0.01; ***p < 0.001.

^a^Age decline is calculated by dividing beta estimate from the % HbA1c measure by the beta association between age and brain outcome from the same model.

### Stratified analysis

We found that results from analyses restricted to middle-aged and older individuals were consistent with results obtained for the entire sample (Supplementary Table S2). In the younger cohort, we found that diabetes status was associated with smaller total brain volume and lower FA (Supplementary Table S3).

### Supplemental regression models

We fit two supplementary regression models using the overall sample, weighted to the target population, with (1) additional adjustments for hypertension and smoking status, and (2) additional adjustments for scanner type (Supplementary Table S4). The results from these models were unchanged relative to the main findings. In models using categorical Hba1c % values, we found that those in the 6.5–7% and >7% groups, but not those in the 5.5–6.5%, had significantly worse outcomes in total brain, lateral ventricles, WMHs, FA, and FW compared to the <5.5% group. The 6.5–7% group also had smaller occipital gray volumes compared to the <5.5% group (Supplementary Table S5).

## Discussion

Using a large cohort of diverse Hispanic/Latino adults, we found that diabetes status and elevated HbA1c % levels were associated with brain volume loss and microstructural abnormalities. Diabetes status, but not prediabetes, was associated with smaller brain volumes, smaller regional gray matter volumes, lower FA, higher FW, increased WMH volume, and larger lateral ventricles volume compared to the no diabetes group. These findings were consistent in the middle-aged and older cohort. In categorical HbA1c % models, we did not find consistent evidence to suggest that those in the >7% group had worse outcomes (larger associations) compared to the 6.5–7% group.

Our results are largely in line with previous evidence. For example, in previous SOL-INCA studies focused on cognitive outcomes, we did not find support for a relationship between prediabetes and worse cognitive function or cognitive decline [30, 31], which is consistent with our mostly lack of findings with the prediabetes group. Data from the Atherosclerosis Risk in Communities Neurocognitive Study (ARIC-NCS; *N* = 1713, mean age 75) suggest that, compared to those without diabetes, individuals with diabetes (35% of cohort) and HbA1c % levels ≥7% had smaller brain volumes and larger WMH volumes [15]. Notably, those with diabetes but HbA1c % <7% were not significantly different compared to the no diabetes group. Findings from the Women’s Health Initiative Magnetic Resonance Imaging Studies (WHIMS-MRI; *N* = 1366; ages 72–89) also indicate that women with diabetes (10% of individuals) had smaller gray matter volumes and greater ischemic load (i.e., infarct volume) compared to women without diabetes [14]. Lastly, the MEMENTO study (*n* = 2288 individuals; mean age 68) found associations between diabetes (11% of individuals) and a neurodegeneration composite, but not with an SVD composite [17]. Although our cohort (mean age 64) was younger relative to these three studies, our cohort had the second largest diabetes prevalence (~25%) and the largest combined prevalence of diabetes and prediabetes (~70%). When we compare the findings based on discrete classifications of HbA1c%, we find that those with values exceeding 7% had worse total brain and WMHs outcomes compared to those with HbA1c < 5.5%. Unlike the ARIC study [15], we found that even those with values 6.5–7% had worse brain outcomes compared to the <5.5% group. These findings suggest that Hispanic/Latino individuals could be at an increased risk of SVD and neurodegeneration compared to predominantly white populations. These estimates, while small, represent an overall large effect of diabetes on ADRD burden in the overall Hispanic/Latino population. The effects of white matter measures on cognition, unless severe, have been shown to be consistent, albeit relatively small [32]. WMHs are also a risk factor for mortality and stroke [33]. While the effects of diabetes on cognition are important, these findings provide evidence for small vessel disease and risk for cerebrovascular sequalae. These findings also build up on previous diabetes work we have performed in this population. We found that diabetes is associated with both cognitive decline and increased risk for mild cognitive impairment [30]. We also found that elevated HbA1c % was associated with accelerated cognitive decline [31]. These results provide possible mechanisms for future study whereby diabetes could lead to these cognitive changes in this population: future work should use mediation models to clarify the role of these brain differences in cognition.

In line with our first hypothesis, we found that diabetes was associated with white matter damage across three measures (WMH volume, FW, FA) in both the overall and middle-aged and older population. WMHs represent white matter lesions [34] and are a risk factor for stroke [35] and dementia [36]. Notably, a recent meta-analysis has shown that WMH is a longitudinal predictor for cognitive decline in cognitively normal populations [37]. As Raja et al. noted, WMH burden also increases with age, and thus DTI measures can help distinguish pathological changes from age-related changes in white matter [38]. Diffusion MRI may also show evidence for white matter damage in normal-appearing white matter (i.e., no WMHs) and thus is seen as an early marker of white matter damage [39–41]. Lastly, DTI measures may be more sensitive to SVD pathology compared to traditional AD (Alzheimer’s Disease) pathology [42], and there is some evidence that DTI measures may predict cognitive changes even in normal-appearing white matter [43]. Our findings suggest that diabetes is associated with both micro and macro white matter lesions and provide evidence for white matter microstructural atrophy in diabetes. Our results with continuous HbA1c % suggest that hyperglycemia, regardless of diabetes status, is associated with worse white matter integrity. In the subgroup of young individuals ages 35–49, we found that diabetes status was associated with higher FA but not WMHs or FW, which suggests there may be early changes in normal-appearing white matter. DTI could potentially be used to track disease progression in individuals with diabetes, even at an early age.

Diabetes could lead to SVD through several mechanisms, including inflammation and endothelial dysfunction [34]. Both hyperglycemia and hyperinsulinemia could alter endothelial function through changes in vasodilating factors such as endothelial nitric oxide synthase [44]. This could explain our results with HbA1c %. Lastly, other vascular sequelae of diabetes, such as atherosclerosis, have also been found to increase ADRD risk [45]. A 2021 review showed that diabetes medication can be effective in reducing microvascular and macrovascular complications [46]. Hispanic/Latino individuals have high rates of uninsurance, and these rates are much higher in individuals 64 and younger due to Medicare [47]. Therefore, increasing diabetes awareness and treatment in Hispanic/Latino populations could be useful for reducing ADRD and SVD risk, and thus, future work should further examine the role of diabetes medication and intervention in ADRD risk.

Our second hypothesis that diabetes would relate to MRI markers of neurodegeneration was partially supported. We found that diabetes status was associated with smaller total brain volumes and larger lateral ventricles, but not with total gray matter or hippocampal volumes. While not as robust as the medial temporal lobe or the hippocampus, both brain volumes and lateral ventricles are seen as markers for Alzheimer’s Disease [48]. Alzheimer’s Disease has been referred to as “type 3 diabetes” because of shared biochemical changes with diabetes, such as brain insulin resistance [49]. Insulin plays key roles in both neurons and glia, including neurogenesis, glucose uptake, and synaptic plasticity. Thus, insulin resistance may lead to increased apoptosis, decreased synaptic plasticity, and eventually cognitive deficits. However, Arnold et al. noted that despite similarities between AD and diabetes, evidence linking diabetes to pathological markers of AD (amyloid and tau) has been mixed or null [13]. Most of these studies were performed on individuals with AD or MCI diagnosis, and thus do not account for early disease progression. Our findings show partial evidence for AD-related changes in individuals with diabetes and hyperglycemia; however, whether these brain changes were due to vascular factors or not is unknown. We have shown, in previous SOL-INCA MRI work, that presence of infarcts was associated with smaller total brain and total gray matter volume [50]. Therefore, further research should include markers of AD (e.g., amyloid, tau) and neurodegeneration (e.g., neurofilament light) to better distinguish between AD, mixed, or non-AD pathology.

This study has several strengths and limitations. We used community-representative data from a deeply characterized Hispanic/Latino cohort in four regions with a high concentration of diverse Hispanic/Latino individuals. Our results provide important information regarding diabetes and brain outcomes in this diverse population. Our study also included multiple MRI modalities, which allowed us to better characterize both micro and macro brain structure. This study also has a few limitations. First, diabetes assessment was performed 10 years prior to MRI acquisition. However, this time difference in the context of this work suggests that earlier life exposure impacts downstream brain outcomes, and thus, early diabetes interventions could be beneficial for brain health. Future work should examine longitudinal changes in diabetes and their effects on brain outcomes. In this study, we did not consider cognitive status in the tested mechanisms. In future work, we will leverage the design of SOL-INCA MRI and test possible differential outcomes by mild cognitive impairment status. This will allow us to consider whether the mechanisms linking diabetes and MRI may differ between individuals with normal cognition and those with preclinical states of disease. We also did not differentiate between type 1 diabetes and type 2 diabetes or assessed diabetes duration, but given low prevalence of type 1 diabetes, it should not significantly affect findings. We also did not include comprehensive control of behavioral risk factors in the models and chose a parsimonious presentation. We ran models that adjusted for hypertension and smoking, and the results from these models were qualitatively and quantitatively equivalent to results presented here. There are several additional factors (e.g., sporadic small vessel disease or multiple sclerosis) that could affect SVD outcomes that were not available to us through this study and were not accounted for in our analysis. Additionally, we used the American Diabetes Association criteria, which includes use of diabetes medication, to classify individuals as having diabetes. Some diabetes medications (e.g., metformin) are used for other conditions (e.g., heart failure), and as such, some people could have been misclassified as having diabetes based on our definition. However, our findings with HbA1c % are consistent with the discrete classification, and thus, we do not believe that this is a substantial issue with our data. Lastly, this is a prospective cohort-based study, and thus susceptible to biases and limitations such as selection bias. We accounted for this by including survey weights in our analysis.

## Conclusion

Our studies highlight possible SVD and neurodegeneration in individuals with diabetes throughout the adult lifespan. Given evidence of SVD, particularly WMHs, on stroke and dementia risk, our findings highlight the need to reduce diabetes burden in this population.

## Supplementary information

Supplementary Materials

## Data Availability

Data used in this study are available only to approved investigators with an accepted research proposal. Investigators interested in obtaining access should submit a proposal at https://sites.cscc.unc.edu/hchs/ and request data access once approved.

## Code availability

All the codes used to generate results are in a private GitLab repository and are available to investigators who collaborate with SOL-INCA. Investigators interested in obtaining code access should submit a proposal at https://sites.cscc.unc.edu/hchs/. Code can be made available to those with approved proposals.
