# Supplementary material for: Diabetes, hyperglycemia, and brain MRI biomarkers: results from SOL-INCA MRI study
Source: Nutr Diabetes. 2026 Apr 7;16:12. doi: 10.1038/s41387-026-00415-z (PMC13172035; doi:10.1038/s41387-026-00415-z)
Supplement: Supplementary file 1 — Supplementary Materials [file 41387_2026_415_MOESM1_ESM.pdf]

Supplemental Figure S1: Population derivation flowchart

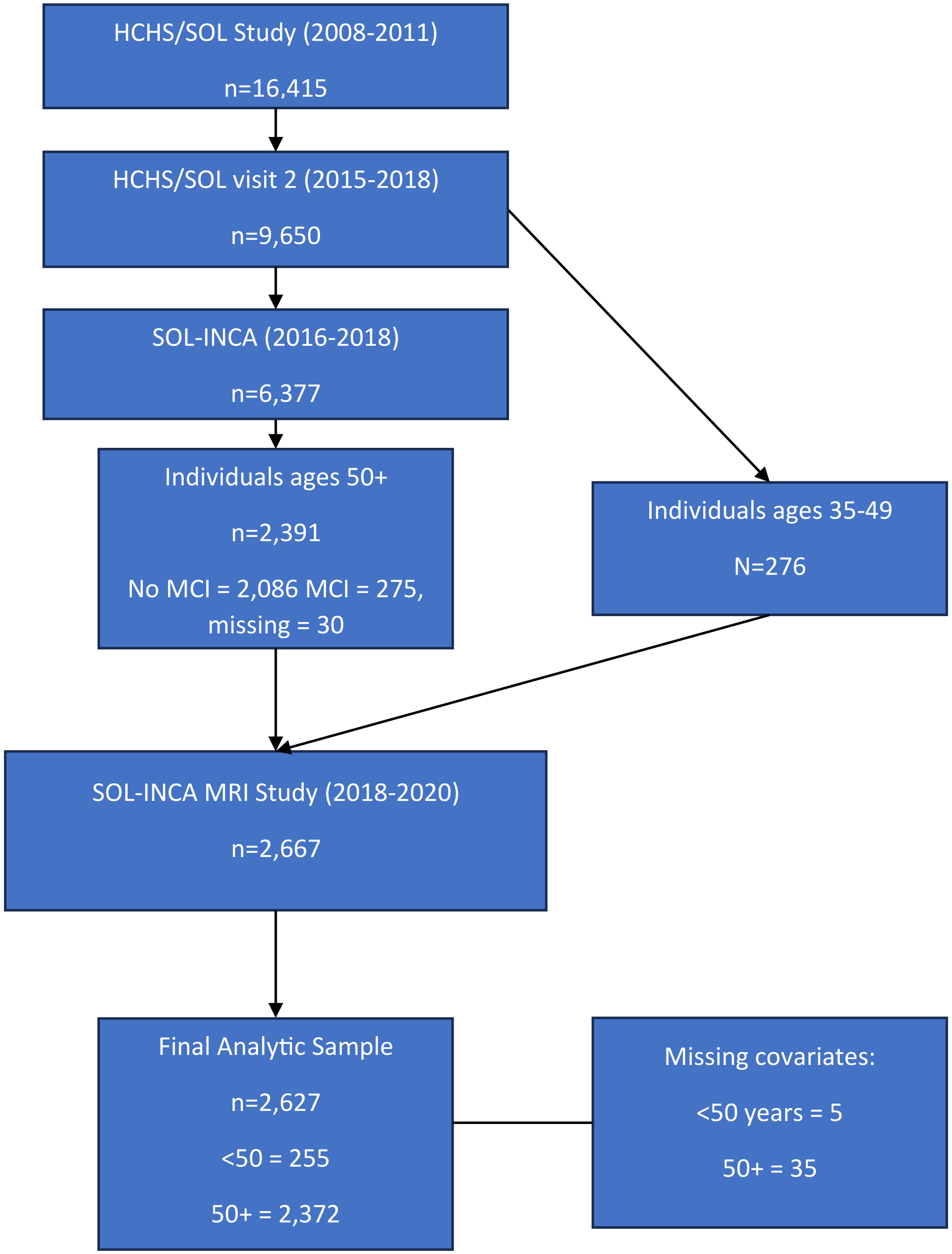

Notes: HCHS/SOL = Hispanic Community Health Study/Study of Latinos; SOL-INCA = Study of Latinos-Investigation of Neurocognitive Aging; MRI= magnetic resonance imaging

Supplemental Methods S1: MRI processing pipeline

Skull stripping was performed with a convolutional neural net model pipeline developed by Fletcher et al.(1) After visual checks, images were registered using a nonlinear cubic B-spline deformation into minimal deformation template synthetic images.(2) Non-uniformity correction was performed using a template-based method, which allowed us to correct for differences in image intensity across individuals.(3)

Gray matter volumes. We estimated gray matter volumes using Expectation-Maximization (EM) algorithm,(4) which consists of both an estimation step and maximization step.(5) An initial space was created using a template of previously segmented images. We used these images to estimate mean and standard deviation of image intensities (estimation step), which were then used as parameters in the EM model (maximization step). Output form this gaussian model was further processed with a Markov Random Field model to generate more robust maps.(4) We measured hippocampal volume using a diffeomorphic approach(6) followed by a mask developed by the AEDC-ADNI group to standardize measures across samples.(7)

WMHs volume was calculated using FLAIR and 3D T1 images. We processed these images using a Bayesian probability structure.(8) A prior probability map and a threshold of 3.5 standard deviations was used to create a binary WMHs mask. Lastly, we transformed segmented WMHs images back to native space to measure WMHs volume.

DTI. FA and FW were calculated using diffusion tensor imaging data. We used the FMRIB Software library toolkit (FSL),(9) which allowed us to also correct for head movements and distortions from currents. To generate an FW fraction map, we used a diffusion

tensor to measure both fractional volume and FW at each voxel. To generate a FA map, we used the DTFIT function from the FSL toolkit and then registered using the FSL FA template space.(10) We used a white matter mask (0.3 threshold) to reduce noise from cerebrospinal fluid.(11) To generate a global measure of FA and FW, we superimposed white matter masks and averaged the score at each white matter voxel.

1. Fletcher E, DeCarli C, Fan AP, Knaack A. Convolutional neural net learning can achieve production-level brain segmentation in structural magnetic resonance imaging. Frontiers in Neuroscience. 2021;15:683426.
2. Kochunov P, Lancaster JL, Thompson P, Woods R, Mazziotta J, Hardies J, et al. Regional spatial normalization: toward an optimal target. Journal of computer assisted tomography. 2001;25(5):805-16.
3. Fletcher E, Carmichael O, DeCarli C, editors. MRI non-uniformity correction through interleaved bias estimation and B-spline deformation with a template. 2012 Annual International Conference of the IEEE Engineering in Medicine and Biology Society; 2012: IEEE.
4. Fletcher E, Singh B, Harvey D, Carmichael O, DeCarli C, editors. Adaptive image segmentation for robust measurement of longitudinal brain tissue change. 2012 annual international conference of the ieee engineering in medicine and biology society; 2012: IEEE.
5. Moon TK. The expectation-maximization algorithm. IEEE Signal processing magazine. 1996;13(6):47-60.
6. Vercauteren T, Pennec X, Perchant A, Ayache N, editors. Non-parametric diffeomorphic image registration with the demons algorithm. International Conference on Medical Image Computing and Computer-Assisted Intervention; 2007: Springer.
7. Boccardi M, Bocchetta M, Apostolova LG, Barnes J, Bartzokis G, Corbetta G, et al. Delphi definition of the EADC-ADNI harmonized protocol for hippocampal segmentation on magnetic resonance. Alzheimer's & Dementia. 2015;11(2):126-38.
8. DeCarli C, Miller B, Swan G, Reed T, Wolf P, Garner J, et al. Predictors of brain morphology for the men of the NHLBI twin study. Stroke. 1999;30(3):529-36.
9. Jenkinson M, Beckmann CF, Behrens TE, Woolrich MW, Smith SM. Fsl. Neuroimage. 2012;62(2):782-90.
10. Smith SM, Kindlmann G, Jbabdi S. Cross-subject comparison of local diffusion MRI parameters. Diffusion MRI: Elsevier; 2014. p. 209-39.
11. Baykara E, Gesierich B, Adam R, Tuladhar AM, Biesbroek JM, Koek HL, et al. A novel imaging marker for small vessel disease based on skeletonization of white matter tracts and diffusion histograms. Annals of neurology. 2016;80(4):581-92.

Supplemental Table S1: Unweighted median and interquartile range of continuous measures

	**35-49 Years**	**50+ years**	**Total**
B**MI median (IQR)**	28.62 (8.11)	29.19 (6.42)	29.10 (6.66)
**HbA1c %**	5.40 (0.40)	5.70 (0.60)	5.70 (0.70)

Notes: BMI = body mass index, IQR = interquartile range. Median values and interquartile range (in parenthesis) are presented.

Supplemental Figure S2: Associations between HbA1c percentage and brain outcomes (marginal estimates and 95% confidence intervals, N=2,627).

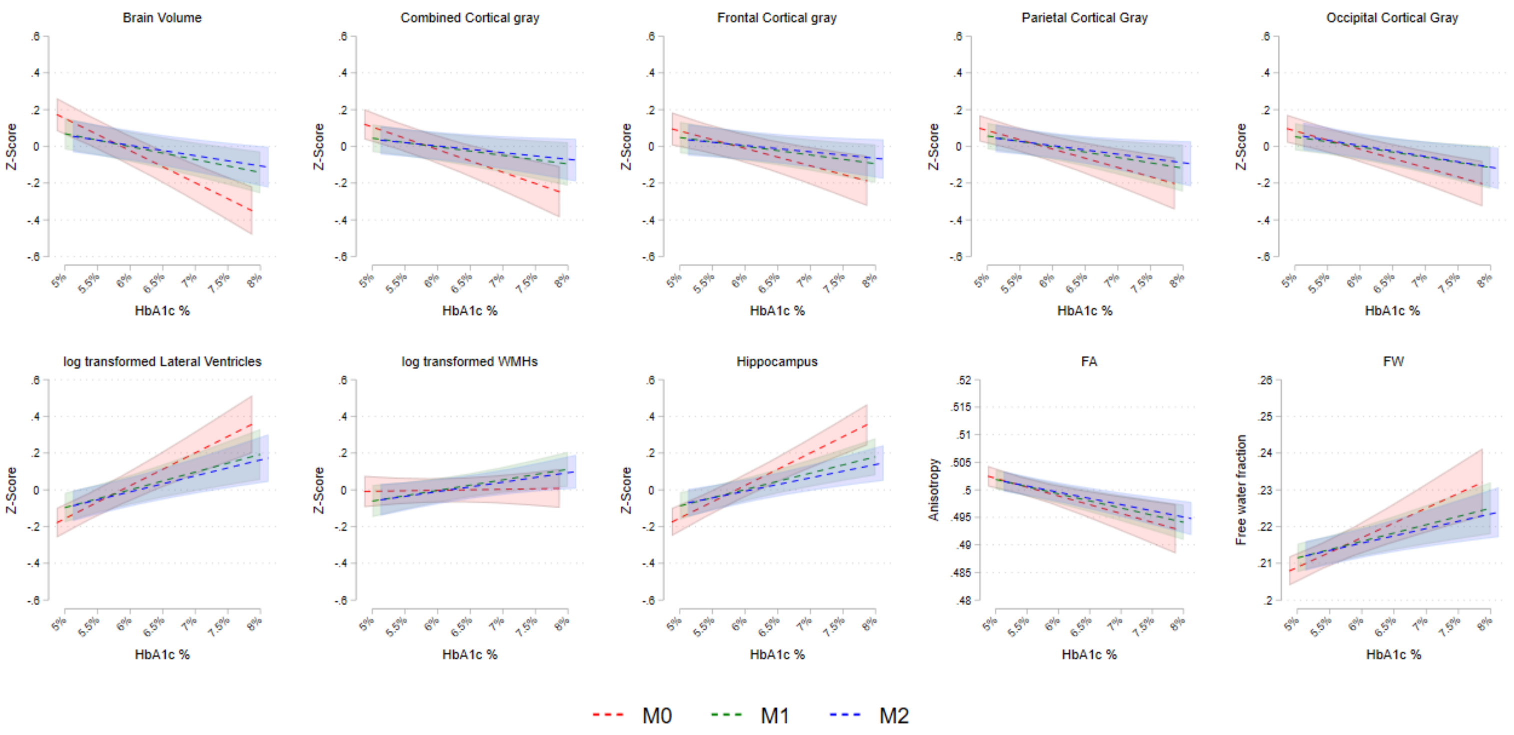

Note: WMH = white matter hyperintensities; FA = free water; FW = free water; all measures, except for FA and FW, are standardized and residualized for cranial volume. Lateral ventricles and WMHs were additionally log transformed before residualization

M0 model includes time between visits.

M1 model additionally includes continuous age, sex (Male, Female), Hispanic/Latino background (South American, Central American, Mexican, Cuban, Puerto Rican, and Dominican), a trichotomous indicator for education (less than high school, high school or equivalent, more than high school), and a categorical measure for testing center (San Diego, Miami, Chicago, Bronx),

M2 additionally includes continuous body mass index, and a four-category indicator for physical activity (Inactive, low activity, medium activity, high activity).

Supplemental Table S2: Associations between diabetes status and brain outcomes in middle-aged and older participants (N=2,372).

	**Brain Volume**		**Combined Gray**		**Frontal Gray**	
	**b [95% CI]**	**b [95% CI]**	**b [95% CI]**	**b [95% CI]**	**b [95% CI]**	**b [95% CI]**
	**M1**	**M2**	**M1**	**M2**	**M1**	**M2**
**No Diabetes**	ref	ref	ref	ref	ref	ref
**Prediabetes**	-0.006 [-0.104;0.092]	0.003 [-0.093;0.098]	0.031 [-0.068;0.130]	0.044 [-0.054;0.142]	0.013 [-0.089;0.115]	0.020 [-0.081;0.121]
**Diabetes**	-0.214*** [-0.324;-0.104]	-0.188*** [-0.296;-0.079]	-0.185** [-0.320;-0.050]	-0.149* [-0.285;-0.013]	-0.239** [-0.382;-0.095]	-0.211** [-0.349;-0.073]

	**Occipital Gray**		**Temporal Gray**		**Parietal Gray**	
	**b [95% CI]**	**b [95% CI]**	**b [95% CI]**	**b [95% CI]**	**b [95% CI]**	**b [95% CI]**
	**M1**	**M2**	**M1**	**M2**	**M1**	**M2**
**No Diabetes**	ref	ref	ref	ref	ref	ref
**Prediabetes**	0.004 [-0.137;0.145]	0.005 [-0.139;0.150]	0.001 [-0.108;0.109]	0.003 [-0.104;0.110]	0.080 [-0.043;0.203]	0.109 [-0.013;0.231]
**Diabetes**	-0.189* [-0.350;-0.028]	-0.183* [-0.349;-0.017]	0.059 [-0.070;0.188]	0.062 [-0.072;0.197]	-0.116 [-0.281;0.050]	-0.046 [-0.209;0.117]

	**Lateral Ventricles**		**Hippocampus**		**WMH**	
	**b [95% CI]**	**b [95% CI]**	**b [95% CI]**	**b [95% CI]**	**b [95% CI]**	**b [95% CI]**
	**M1**	**M2**	**M1**	**M2**	**M1**	**M2**
**No Diabetes**	ref	ref	ref	ref	ref	ref
**Prediabetes**	0.086 [-0.018;0.190]	0.075 [-0.028;0.179]	0.049 [-0.087;0.186]	0.032 [-0.099;0.164]	0.086 [-0.018;0.190]	0.075 [-0.028;0.179]
**Diabetes**	0.333*** [0.213;0.453]	0.302*** [0.178;0.426]	0.141 [-0.046;0.328]	0.100 [-0.084;0.284]	0.333*** [0.213;0.453]	0.302*** [0.178;0.426]

	**FA**		**FW**	
	**b [95% CI]**	**b [95% CI]**	**b [95% CI]**	**b [95% CI]**
	**M1**	**M2**	**M1**	**M2**
**No Diabetes**	ref	ref	ref	ref
**Prediabetes**	-0.001 [-0.004;0.001]	-0.001 [-0.003;0.002]	-0.003 [-0.007;0.002]	-0.003 [-0.007;0.002]
**Diabetes**	-0.009*** [-0.012;-0.005]	-0.008*** [-0.011;-0.004]	0.016*** [0.008;0.024]	0.016*** [0.009;0.024]

Notes: WMH = white matter hyperintensities; FA = fractional anisotropy; FW = free water.
M1 model includes time between visits, continuous age, sex (Male, Female), Hispanic/Latino background (South American, Central American, Mexican, Cuban, Puerto Rican, and Dominican), a trichotomous indicator for education (less than high school, high school or equivalent, more than high school), and a categorical measure for testing center (San Diego, Miami, Chicago, Bronx).
M2 additionally includes continuous body mass index, and a four-category indicator for physical activity (Inactive, low activity, medium activity, high activity).
* = $p < 0.05$; ** = $p < 0.01$; *** = $p < 0.001$. Differences relative to reference group (No diabetes)

Supplemental Table S3: Associations between diabetes status and brain outcomes in individuals ages 35-49 (N=255).

	**Brain Volume**		**Combined Gray**		**Frontal Gray**	
	**b [95% CI]**	**b [95% CI]**	**b [95% CI]**	**b [95% CI]**	**b [95% CI]**	**b [95% CI]**
	**M1**	**M2**	**M1**	**M2**	**M1**	**M2**
**No Diabetes**	ref	ref	ref	ref	ref	ref
**Prediabetes**	-0.115 [-0.293;0.062]	-0.078 [-0.256;0.100]	-0.115 [-0.468;0.238]	-0.079 [-0.395;0.236]	-0.143 [-0.468;0.181]	-0.063 [-0.364;0.238]
**Diabetes**	-0.365* [-0.666;-0.064]	-0.323* [-0.618;-0.029]	0.142 [-0.304;0.589]	0.209 [-0.298;0.717]	-0.033 [-0.405;0.338]	0.151 [-0.284;0.586]
	**Occipital Gray**		**Temporal Gray**		**Parietal Gray**	
	**b [95% CI]**	**b [95% CI]**	**b [95% CI]**	**b [95% CI]**	**b [95% CI]**	**b [95% CI]**
	**M1**	**M2**	**M1**	**M2**	**M1**	**M2**
**No Diabetes**	ref	ref	ref	ref	ref	ref
**Prediabetes**	-0.053 [-0.309;0.204]	-0.053 [-0.317;0.211]	-0.122 [-0.469;0.225]	-0.113 [-0.421;0.196]	0.036 [-0.330;0.403]	0.010 [-0.332;0.353]
**Diabetes**	0.330 [-0.052;0.712]	0.329 [-0.091;0.750]	0.225 [-0.239;0.689]	0.243 [-0.333;0.820]	-0.012 [-0.524;0.500]	-0.121 [-0.655;0.414]
	**Lateral Ventricles**		**Hippocampus**		**WMH**	
	**b [95% CI]**	**b [95% CI]**	**b [95% CI]**	**b [95% CI]**	**b [95% CI]**	**b [95% CI]**
	**M1**	**M2**	**M1**	**M2**	**M1**	**M2**
**No Diabetes**	ref	ref	ref	ref	ref	ref
**Prediabetes**	0.051 [-0.202;0.304]	0.083 [-0.182;0.348]	0.319* [0.037;0.601]	0.297* [0.008;0.586]	-0.212 [-0.473;0.049]	-0.230 [-0.500;0.041]
**Diabetes**	0.371 [-0.036;0.777]	0.489* [0.072;0.906]	0.124 [-0.199;0.448]	0.032 [-0.366;0.429]	0.400 [-0.004;0.804]	0.275 [-0.094;0.643]
	**FA**		**FW**			
	**b [95% CI]**	**b [95% CI]**	**b [95% CI]**	**b [95% CI]**		
	**M1**	**M2**	**M1**	**M2**		
**No Diabetes**	ref	ref	ref	ref		
**Prediabetes**	-0.003 [-0.007;0.002]	-0.003 [-0.008;0.001]	0.001 [-0.006;0.007]	0.001 [-0.005;0.008]		
**Diabetes**	-0.015*** [-0.021;-0.009]	-0.016*** [-0.023;-0.009]	0.008 [-0.004;0.019]	0.011 [-0.001;0.023]		

Notes: WMH = white matter hyperintensities; FA = free water; FW = free water.

M1 model includes time between visits, continuous age, sex (Male, Female), Hispanic/Latino background (South American, Central American, Mexican, Cuban, Puerto Rican, and Dominican), a trichotomous indicator for education (less than high school, high school or equivalent, more than high school), and a categorical measure for testing center (San Diego, Miami, Chicago, Bronx).
M2 additionally includes continuous body mass index, and a four-category indicator for physical activity (Inactive, low activity, medium activity, high activity).
* = $p < 0.05$; ** = $p < 0.01$; *** = $p < 0.001$. Differences relative to reference group (No diabetes)

Supplemental Table S4: Associations between diabetes status and brain outcomes.

	Total Brain		Combined Gray		Frontal Gray	
	**b [95% CI]**	**b [95% CI]**	**b [95% CI]**	**b [95% CI]**	**b [95% CI]**	**b [95% CI]**
	**M3**	**M4**	**M3**	**M4**	**M3**	**M4**
**No diabetes**	ref	ref	ref	ref	ref	ref
**Prediabetes**	0.008 [-0.086;0.102]	0.005 [-0.087;0.096]	0.048 [-0.056;0.152]	0.056 [-0.039;0.151]	0.025 [-0.080;0.131]	0.033 [-0.069;0.135]
**Diabetes**	-0.206*** [-0.322;-0.090]	-0.224*** [-0.339;-0.108]	-0.143* [-0.281;-0.006]	-0.114 [-0.235;0.006]	-0.192** [-0.333;-0.051]	-0.164* [-0.289;-0.039]

	Occipital Gray		Temporal Gray		Parietal Gray	
	**b [95% CI]**	**b [95% CI]**	**b [95% CI]**	**b [95% CI]**	**b [95% CI]**	**b [95% CI]**
	**M3**	**M4**	**M3**	**M4**	**M3**	**M4**
**No diabetes**	ref	ref	ref	ref	ref	ref
**Prediabetes**	0.019 [-0.109;0.146]	0.028 [-0.097;0.152]	-0.003 [-0.107;0.101]	-0.011 [-0.112;0.090]	0.108 [-0.013;0.229]	0.124* [0.016;0.232]
**Diabetes**	-0.181* [-0.332;-0.031]	-0.143 [-0.288;0.001]	0.074 [-0.062;0.211]	0.063 [-0.070;0.195]	-0.076 [-0.241;0.088]	-0.046 [-0.187;0.095]

	Lateral Ventricles		Hippocampus		WMHs	
	**b [95% CI]**	**b [95% CI]**	**b [95% CI]**	**b [95% CI]**	**b [95% CI]**	**b [95% CI]**
	**M3**	**M4**	**M3**	**M4**	**M3**	**M4**
**No diabetes**	ref	Ref	ref	ref	ref	ref
**Prediabetes**	0.024 [-0.087;0.136]	0.012 [-0.099;0.123]	0.108 [-0.012;0.228]	0.087 [-0.029;0.204]	0.010 [-0.088;0.107]	0.014 [-0.078;0.107]
**Diabetes**	0.298*** [0.129;0.467]	0.288*** [0.128;0.449]	0.171 [-0.004;0.345]	0.130 [-0.047;0.307]	0.266*** [0.138;0.394]	0.271*** [0.149;0.394]

	FA		FW	
	**b [95% CI]**	**b [95% CI]**	**b [95% CI]**	**b [95% CI]**
	**M3**	**M4**	**M3**	**M4**
**No diabetes**	ref	ref	ref	ref
**Prediabetes**	-0.001 [-0.003;0.001]	-0.001 [-0.003;0.001]	-0.004* [-0.008;-0.001]	-0.003 [-0.007;0.001]
**Diabetes**	-0.008*** [-0.011;-0.005]	-0.008*** [-0.011;-0.005]	0.013*** [0.006;0.019]	0.017*** [0.010;0.023]

Notes: WMH = white matter hyperintensities; FA = free water; FW = free water.

M3: model includes continuous age, sex (Male, Female), Hispanic/Latino background (South American, Central American, Mexican, Cuban, Puerto Rican, and Dominican), and a trichotomous indicator for education (less than high school, high school or equivalent, more than high school), a categorical measure for testing center (San Diego, Miami, Chicago, Bronx), continuous body mass index, a

four-category indicator for physical activity (Inactive, low activity, medium activity, high activity), smoking status, and hypertension (no hypertension, hypertension)
M4: model includes continuous age, sex (Male, Female), Hispanic/Latino background (South American, Central American, Mexican, Cuban, Puerto Rican, and Dominican), and a trichotomous indicator for education (less than high school, high school or equivalent, more than high school), a categorical measure for testing center (San Diego, Miami, Chicago, Bronx), continuous body mass index, a four-category indicator for physical activity (Inactive, low activity, medium activity, high activity), and scanner type
* = $p < 0.05$; ** = $p < 0.01$; *** = $p < 0.001$. Differences relative to reference group (No diabetes)

Supplemental Table S5: Associations between Hba1c % thresholds and brain outcomes

	**Total Brain**		**Combined Gray**		**Frontal Gray**	
	**b [95% CI]**	**b [95% CI]**	**b [95% CI]**	**b [95% CI]**	**b [95% CI]**	**b [95% CI]**
	**M1**	**M2**	**M1**	**M2**	**M1**	**M2**
**<5.5 %**	ref	ref	ref	ref	ref	ref
**5.5%-6.5%**	0.106 [-0.005;0.218]	0.118* [0.006;0.229]	0.057 [-0.055;0.169]	0.072 [-0.033;0.178]	0.079 [-0.035;0.193]	0.098 [-0.012;0.207]
**6.5%-7%**	-0.279** [-0.489;-0.068]	-0.260* [-0.466;-0.054]	-0.242 [-0.507;0.023]	-0.203 [-0.471;0.066]	-0.237* [-0.437;-0.038]	-0.193 [-0.387;0.002]
**>7%**	-0.307*** [-0.464;-0.149]	-0.278*** [-0.443;-0.113]	-0.136 [-0.299;0.027]	-0.103 [-0.267;0.062]	-0.129 [-0.303;0.045]	-0.087 [-0.261;0.087]
	**Occipital Gray**		**Temporal Gray**		**Parietal Gray**	
	**b [95% CI]**	**b [95% CI]**	**b [95% CI]**	**b [95% CI]**	**b [95% CI]**	**b [95% CI]**
	**M1**	**M2**	**M1**	**M2**	**M1**	**M2**
**<5.5 %**	ref	ref	ref	ref	ref	ref
**5.5%-6.5%**	-0.118 [-0.258;0.023]	-0.118 [-0.261;0.024]	0.077 [-0.050;0.205]	0.079 [-0.047;0.204]	0.085 [-0.045;0.215]	0.105 [-0.020;0.231]
**6.5%-7%**	-0.375* [-0.702;-0.047]	-0.365* [-0.698;-0.032]	0.183 [-0.065;0.430]	0.185 [-0.072;0.442]	-0.277 [-0.645;0.092]	-0.230 [-0.603;0.144]
**>7%**	-0.159 [-0.327;0.008]	-0.164 [-0.336;0.008]	0.069 [-0.088;0.226]	0.076 [-0.081;0.233]	-0.174 [-0.364;0.016]	-0.134 [-0.323;0.054]
	**Lateral Ventricles**		**Hippocampus**		**WMHs**	
	**b [95% CI]**	**b [95% CI]**	**b [95% CI]**	**b [95% CI]**	**b [95% CI]**	**b [95% CI]**
	**M1**	**M2**	**M1**	**M2**	**M1**	**M2**
**<5.5 %**	ref	Ref	ref	ref	ref	ref
**5.5%-6.5%**	-0.065 [-0.173;0.043]	-0.081 [-0.190;0.027]	0.066 [-0.086;0.218]	0.039 [-0.114;0.191]	0.123* [0.008;0.238]	0.104 [-0.007;0.215]
**6.5%-7%**	0.481* [0.109;0.852]	0.460* [0.110;0.810]	-0.125 [-0.383;0.132]	-0.185 [-0.447;0.077]	0.343*** [0.170;0.516]	0.293*** [0.123;0.463]
**>7%**	0.363*** [0.187;0.540]	0.334*** [0.162;0.506]	0.184 [-0.008;0.376]	0.142 [-0.052;0.336]	0.378*** [0.211;0.546]	0.347*** [0.180;0.514]
	**FA**		**FW**			
	**b [95% CI]**	**b [95% CI]**	**b [95% CI]**	**b [95% CI]**		
	**M1**	**M2**	**M1**	**M2**		
**<5.5 %**	ref	ref	ref	ref		
**5.5%-6.5%**	-0.001 [-0.003;0.002]	-0.000 [-0.003;0.002]	-0.003 [-0.008;0.002]	-0.003 [-0.008;0.002]		
**6.5%-7%**	-0.012*** [-0.017;-0.006]	-0.011*** [-0.016;-0.006]	0.025*** [0.012;0.038]	0.025*** [0.012;0.038]		
**>7%**	-0.010*** [-0.014;-0.005]	-0.009*** [-0.014;-0.004]	0.018*** [0.009;0.028]	0.019*** [0.009;0.028]		

Notes: WMH = white matter hyperintensities; FA = free water; FW = free water.

M1 model includes continuous age, sex (Male, Female), Hispanic/Latino background (South American, Central American, Mexican, Cuban, Puerto Rican, and Dominican), a trichotomous indicator for education (less than high school, high school or equivalent, more than high school), and a categorical measure for testing center (San Diego, Miami, Chicago, Bronx).
M2 additionally includes continuous body mass index, and a four-category indicator for physical activity (Inactive, low activity, medium activity, high activity).
* = $p < 0.05$; ** = $p < 0.01$; *** = $p < 0.001$. Differences relative to reference group (No diabetes)

Supplemental Figure S3: Histogram of outcomes

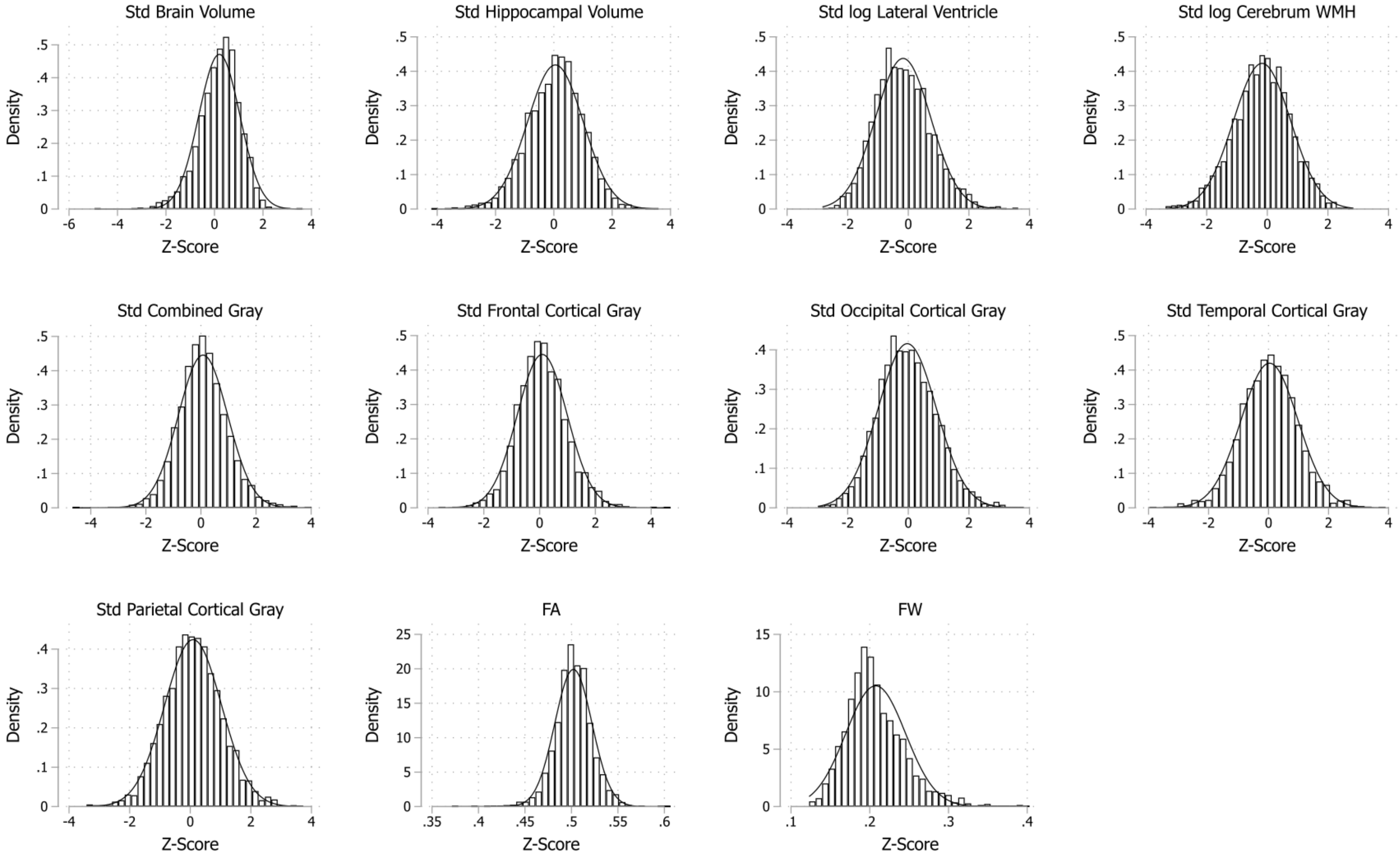

Notes:

Std = standardized, FA = fractional anisotropy, FW = free water, WMH = white matter hyperintensities
